# Novel protein from larval sponge cells, ilborin, is related to energy turnover and calcium binding and is conserved among marine invertebrates

**DOI:** 10.1098/rsob.210336

**Published:** 2022-02-23

**Authors:** Ilya Borisenko, Maria Daugavet, Alexander Ereskovsky, Andrey Lavrov, Olga Podgornaya

**Affiliations:** ^1^ Department of Embryology, Faculty of Biology, Saint Petersburg State University, Saint Petersburg, Russia; ^2^ Institute of Cytology, Russian Academy of Sciences, Saint Petersburg, Russia; ^3^ Institut Méditerranéen de Biodiversité et d'Ecologie Marine et Continentale (IMBE), Université d' Aix-Marseille, CNRS, IRD, Marseille, France; ^4^ Evolution of Morphogenesis Laboratory, Koltzov Institute of Developmental Biology of the Russian Academy of Sciences, Moscow, Russia; ^5^ Pertsov White Sea Biological Station, Biological Faculty, Lomonosov Moscow State University, Moscow, Russia

**Keywords:** Porifera, evolution, metamorphosis, transdifferentiation, larva, protein

## Abstract

Sponges (phylum Porifera) are early-branching animals, whose outwardly simple body plan is underlain by a complex genetic repertoire. The transition from a mobile larva to an attached filter-feeding organism occurs by metamorphosis, a process accompanied by a radical change of the body plan and cell transdifferentiation. The continuity between larval cells and adult tissues is still obscure. In a previous study, we have produced polyclonal antibodies against the major protein of the flagellated cells covering the larva of the sponge *Halisarca dujardini*, used them to trace the fate of these cells and shown that the larval flagellated cells transdifferentiate into the choanocytes. In the present work, we identified the sequence of this novel protein, which we named ilborin. A search in the open databases showed that multiple orthologues of the newly identified protein are present in sponges, cnidarians, flatworms, ctenophores and echinoderms, but none of them has been described yet. Ilborin has two conserved domains: triosephosphate isomerase-barrel, which has enzymatic activity against macroergic compounds, and canonical EF-hand, which binds calcium. mRNA of ilborin is expressed in the larval flagellated cells. We suggest that the new protein is involved in the calcium-mediated regulation of energy metabolism, whose activation precedes metamorphosis.

## Graphical abstract

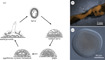

## 1. Background

Cellular activity, mostly migration, during development, regeneration and metamorphosis requires energy. While biochemical mechanisms of energy turnover are well described, its regulation is still obscure. Calcium ions (Ca^2+^) are the most versatile cytoplasmic secondary messenger, which bind to highly conserved calcium-binding domains of proteins and play their role through changes of conformation of the target protein. The role of energy turnover seems obvious and the signalling functions of Ca^2+^ are widely described throughout the animal tree of life [1].

Sponges are a popular subject of studies on molecular evolution and the evolution of animal body plans owing to their ancient branching point in the metazoan lineage. An outwardly simple body plan of sponges is underlain by a complex genetic repertoire. The role of the sponge larva as a step following the choanoblastea in the appearance of multicellularity and the fate of the sponge larval cells in metamorphosis are widely discussed [2–8].

**Figure 1.**
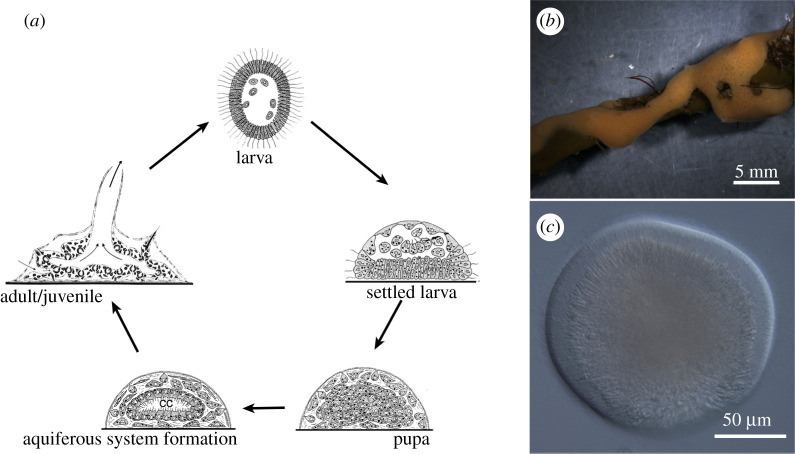
(*a*) Sexual life cycle of marine sponge (adult/juvenile individual, larva, settled larva transforming into pupa, formation of aquiferous system inside the pupa); (*b*) marine sponge *H. dujardini in vivo* (scale bar, 5 mm); (*c*) swimming larva of *Halisarca dujardini* (differential interference contrast microscopy; scale bar, 50 µm).

The adult sponge is a sedentary animal that feeds by filtration. Its body is covered with exopinacoderm (a layer of covering cells, exopinacocytes) and pierced with a network of canals of the aquiferous system (figure 1*a,b*). Water enters the aquiferous system of the sponge through numerous holes (ostia) on the body surface and then passes through the canals and the choanocyte chambers into the atrial cavity and leaves the body through the osculum. This water flow is created by the beating of the flagella of the choanocytes, the cells making up the choanocyte chambers. Choanocytes also trap and phagocytose food particles, give rise to reproductive cells and may participate in fertilization, as in calcareous sponges [9]. Choanocytes are the only cell type with actively beating flagella, though other cells, such as pinacocytes, may bear immotile ones [10]. The space between the layers of the exo-, endopinacoderm and choanoderm is filled with mesohyl, an extracellular matrix. Motile cells may also be present in the mesohyl, but their locomotor activity is limited to lobo/lamello/pseudopodial movement.

A striking example of the ability of adult sponge cells to de- and transdifferentiate is the cell reaggregation process [11,12]. After complete tissue dissociation, individual sponge cells retain their viability and form multicellular aggregates. Initially, these aggregates are loose masses of cells, devoid of any structure. However, they can develop, and in certain cases become a functional sponge [13–16]. The reaggregation process relies on active cell migrations, extensive dedifferentiations (at the initial stages) and transdifferentiations (during the development of the aggregates) [12–15]. A key stage of cell reaggregation is the primmorph, a multicellular aggregate with a continuous external epithelium (exopinacoderm) enveloping an internal mass of mainly dedifferentiated cells. The primmorphs can develop active morphogenetic processes occuring in their inner parts, resulting in the gradual restoration of the anatomical structures of the sponge such as skeleton, mesohyl, aquiferous system, etс.

Sponges reproduce sexually and, in various ways, asexually (by fragmentation, budding and formation of gemmules). Embryonic development, which usually occurs inside the mother's body, ends with the emergence of an actively swimming larva (figure 1*b*). It is covered by flagellated cells, which enable swimmingactivity. Sponge larvae are structurally diverse, with seven types being distinguished [5]. After swimming in plankton, the larva finds a suitable, often specific substrate, attaches to it with its anterior pole and undergoes metamorphosis. As a result, a juvenile is formed, a rhagon with a single choanocyte chamber, which grows and becomes a new sponge.

The embryonic development and metamorphosis of the demosponge *Halisarca dujardini* have been studied in detail at the ultrastructural level [2,17,18]. A natural marker of flagellated larval cells, the S-antigen, was used to trace their fate during metamorphosis. In calcium-free water, the larvae were dissociated into individual cells, the cell suspension was separated by differential centrifugation in a density gradient, and the S-antigen was identified in the flagellated cell fraction with protein electrophoresis. Antibodies to the S-antigen bind specifically to the cytoplasm of the larval flagellar cells. In the course of metamorphosis, the antibodies marked the cells inside the ‘pupa’ (the attached larva) and the choanocytes in the adult sponge [19]. Thus, the continuity between the flagellated cells of the larva and the choanocytes of the young sponge was shown for *H. dujardini*. The major protein marker (S-antigen) has never been identified.

The aim of this work was to identify and to characterize the S-antigen protein. *Halisarca dujardini* transcriptome has been sequenced previously [20]. On sodium dodecyl-sulfate polyacrylamide gel electrophoresis (SDS-PAGE), we found S-antigen in the fraction of flagellated cells based on its molecular weight. The S-antigen band was cut out of the gel and subjected to mass spectrometry (MS) followed by bioinformatics analysis.

We considered the S-antigen identified in our study to be a new protein, since we found no described orthologues in the literature, and we named it ilborin. There are several unidentified homologues of ilborin in the protein databases of different invertebrates. We revealed the domain structure and the expression of ilborin as well as its diversity among Metazoa. We assume that this protein is involved in the energy metabolism regulated by calcium and that its function is conserved in several groups of marine invertebrates.

## 2. Results

### 2.1. Cell separation and protein identification

**Figure 2.**
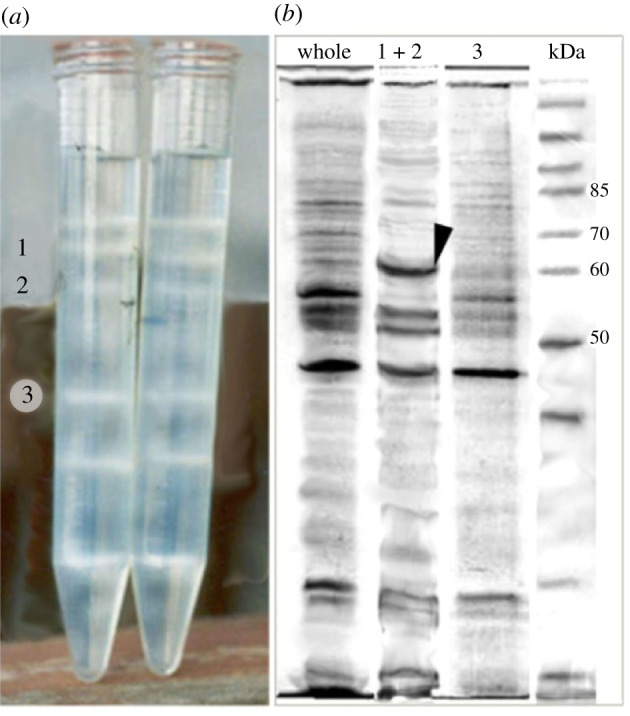
(*a*) Dissociated larval cells separated in Percoll discontinuous gradient. Fractions 1 and 2 are enriched with anterolateral flagellated cells, and fraction 3, with cells of the posterior pole of the larva. Major protein of anterolateral flagellated cells (arrowhead) is absent in posterior cells of fraction 3. (*b*) SDS-PAGE of whole larval proteins (‘whole’ lane) and corresponding fractions.

The *H. dujardini* larva is 120–150 µm in diameter and has a spherical shape with flattened posterior pole (figure 1*c*). Intercellular junctions in Porifera are Ca-dependent, and thus placing the larvae in Ca-free seawater supplemented with a calcium-binding chelator led to the dissociation of the tissue into single cells. A suspension of dissociated larvae cells was separated by using a discontinuous density Percoll gradient. The cell fraction located between 10 and 25% Percoll consists of flagellated cells of the anterolateral surface of the larva (figure 2*a*; [19]). This cell fraction showed four major protein bands on SDS-PAGE (figure 2*b*). The upper major protein band with molecular weight 65–68 kDa corresponded to the S-antigen previously used for antibody production [19]. This band was subjected to trypsin digestion and MS/MS accompanied by searching in proteomic databases based on transcriptome assembly of *H. dujardini* (electronic supplementary material, file S1)*.* The search yielded a 577 aa residue protein sequence. The attempt to find homologous proteins by tBLASTn against the NCBI Nucleotide database showed the lack of reliable similarity with any known proteins. We assumed that the protein was novel and named it ilborin. An additional search in the transcriptome revealed three more paralogues of ilborin, and we indicated them by letters from A to D (GenBank accession numbers MT559266–MT559269). Each of the ilborin transcripts has distinct 5′ and 3′ untranslated regions (electronic supplementary material, file S2). The existence of four transcripts was confirmed by PCR with primers designed to untranslated regions (UTRs). Sequences are supplied in the electronic supplementary material files: full-length transcripts with UTR (electronic supplementary material, file S3), aligned coding DNA sequence (CDS) (electronic supplementary material, file S4), 5′ UTR (electronic supplementary material, file S5) and 3′ UTR (electronic supplementary material, file S6), aligned proteins of four *H. dujardini* ilborins (electronic supplementary material, file S7).

A closer look on the paralogues shows that (i) the nucleotide and putative amino acid differences are distributed throughout the complete sequences of all variants (electronic supplementary material, files S4–S7), (ii) the 5′ UTR sequences of *ilborinA* and *ilborinB* look identical, whereas the 5′ UTR regions of *ilborinC* and *ilborinD* are very different (electronic supplementary material, files S2 and S5); (iii) 3′ UTR sequences of *ilborins* are very different (electronic supplementary material, file S6). Such a structure suggests that we may expect different ilborin paralogues to be encoded by different genes.

### 2.2. Sequence analysis

**Figure 3.**
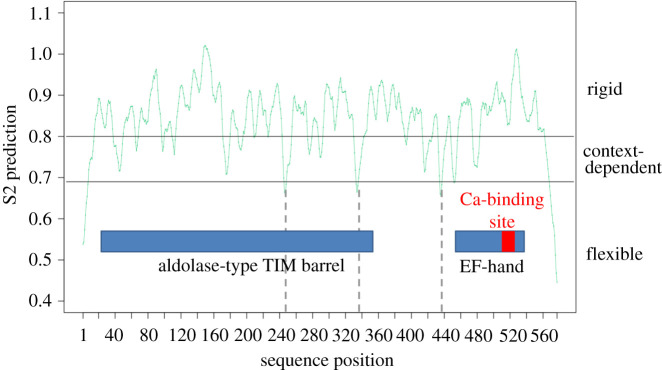
Protein backbone disorder prediction for ilborin A from *Halisarca dujardini*. Blue rectangles show borders of homology to particular superfamilies identified using InterPro [21]. The Ca-binding site is marked by a red box. Regions of maximum flexibility inside the amino acid sequence are marked by dashed lines.

ilborin paralogues (Hdu_ilborin A, B, C, D) share 78% identitical amino acid positions. The four *hduilborin* gene ORFs comprise 577–583 aa residues. Hdu_ilborins are acidic proteins with a mean theoretical molecular mass of 66.386 kDa (table 1). They have no signal peptide targeting them to any special cell compartment or secretion, neither do they have any predicted transmembrane domains. Based on this, Hdu_ilborins were considered to have cytoplasmic localization. Hdu_ilborin A was analysed further as a representative of the four paralogues. Protein backbone disorder prediction showed an overall rigidity of the sequence (S2 > 0.8) with three inner disordered regions (S2 < 0.7; figure 3, dashed line). Two regions belonging to the superfamily aldolase-type triosephosphate isomerase (TIM) barrel (IPR013785; 22–354 aa residues) and EF-hand domain pair (IPR011992; 458–540 aa residues) were predicted (figure 3). The aldolase-type TIM barrel superfamily contains many proteins with enzymatic activity possessing a characteristic beta/alpha barrel fold. Ca-binding proteins usually have an EF-hand domain. A highly conserved Ca-binding site from 514 to 526 aa residues (IPR018247) present in the Hdu_ilborin A EF-hand domain (figure 3) was identified in other invertebrates as well as in the sequence of all three other paralogues. It is described below and in electronic supplementary material, file S8.

**Table 1.** Predicted properties of ilborin paralogues from *Halisarca dujardini*.

	ORF length (aa)	MW (dDa)	(Asp + Glu)	(Arg + Lys)	p*I*
hduilborin A	577	66 536.12	88	77	5.84
hduilborin B	577	66 392.82	86	78	6.03
hduilborin C	586	67 160.8	89	77	5.82
hduilborin D	569	65 453.59	89	67	5.31

### 2.3. Ilborin expression

**Figure 4.**
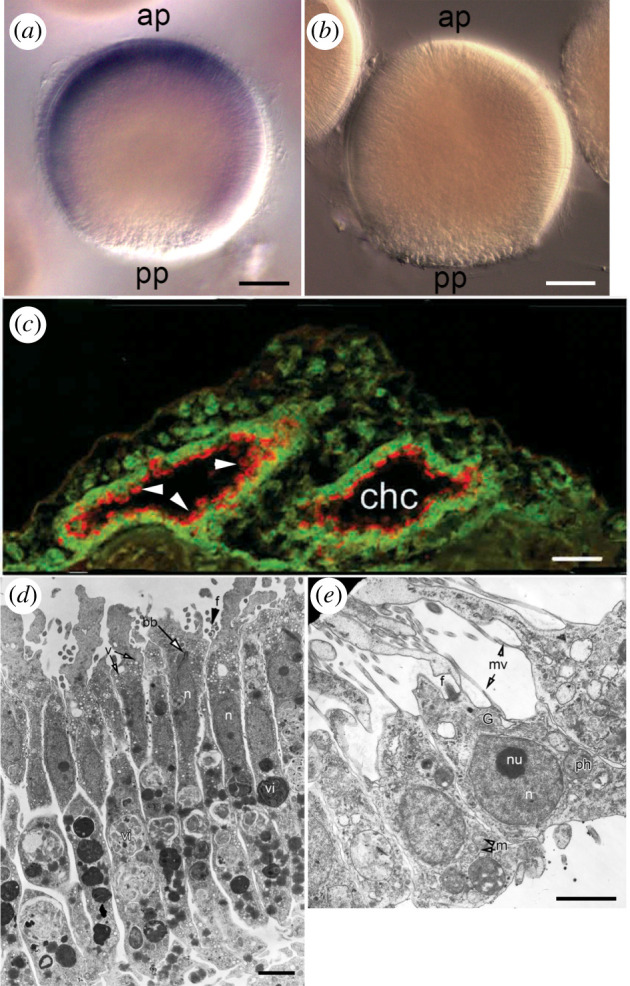
*Ilborin* expression and associated structures. WMISH with antisense (*a*) and sense (*b*, negative control) probes to *ilborin A* on whole larva. ap, anterior pole; pp, posterior pole. (*c*) Paraffin section of juvenile sponge 3 days after settlement, immunostained with antibodies to p68 (ilborin; red) and tubulin (green); arrowheads indicate the apical parts of choanocytes (from [20]). chc, choanocyte chamber formed *de novo*. TEM of larval anterolateral flagellated cells (*d*) and adult choanocytes (*e*). bb, basal body of flagellum; G, Golgi complex; mv, microvilli; m, mitochondria; n, nucleus; nu, nucleolus; v, electron-transparent vesicles; vi, vitelline inclusions. Scale bars: (*a*) and (*b*), 25 µm; (*c*), 10 µm; (*d*,*e*), 2 µm.

The localization of ilborin transcripts in the larval cells and developing primmorphs was examined by whole-mount *in situ* hybridization (WMISH). Specific primers for the 543 bp fragment were designed based on the full-length transcript (see §5). The transcript was observed in flagellated anterolateral cells of the larva, with maximal expression at anterior pole while the cells of the posterior pole seemed to be clear of staining (figure 4*a*). The presence of a corresponding protein in all anterolateral flagellated cells has been shown by antibody detection in a previous work [19]. Digoxigenin-labelled sense RNA probe was used as negative control; no staining was observed in control experiments (figure 4*b*). The staining pattern in WMISH could be explained by a spatio-temporal restriction of translation: protein synthesis in the posterior half stopped, and mRNA was no more detected, but the protein had already been diposed in the cytoplasm. Just after metamorphosis, in the juvenile sponge, ilborin localized in the apical parts of choanocytes (figure 4*c*). In the adult, sponge structures resembling choanocyte chambers strongly bind the ilborin antisense probe (electronic supplementary material, file S9E,F).

**Figure 5.**
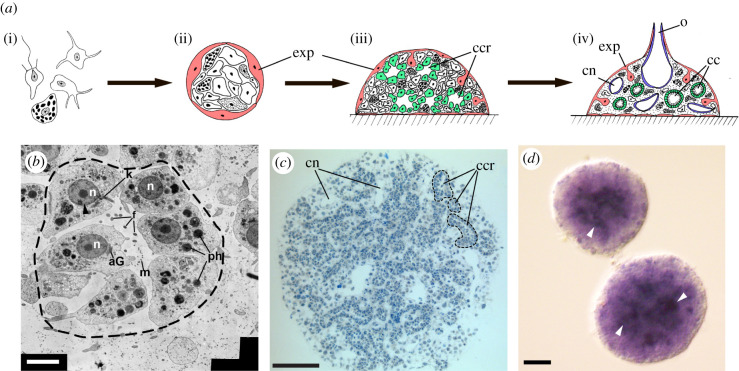
Primmorph development from dissociated sponge cells, and *ilborin* expression. (*a*) Scheme of sponge development from dissociated cells. Cells in suspension (i) make contact and form aggregates (ii) with the outer layer of cells developing in the exopinacoderm. Then, the aggregate attaches to the substrate and inner cells dedifferentiate and differentiate into choanocytes, endopynacocytes and mesohylar cells (iii), which then form the adult sponge body (iv). (*b*) Transmission electron microscopy section of rudimentary choanocyte chamber (outlined with dashed line) consisting of differentiating choanocytes bearing small flagella. (*c*) Semi-thin section of primmorph, demonstrating development of aquiferous system and rudiments of choanocyte chambers (144 h). (*d*) WMISH with *ilborin* probe with developing primmorph (288 h). Main signal, indicated by arrowheads, corresponds to newly formed choanocyte chambers. aG, Golgi apparatus; cc, choanocyte chambers; ccr, rudimentary choanocyte chamber; cn, canal of aquiferous system; exp, exopinacoderm; f, flagella; m, mitochondrion; n, nucleus; o, osculum; ph, phagosomes. Scale bars: (*b*), 100 µm; (*c*), 2 µm; (*d*), 20 μm.

We observed *ilborin* expression in developing primmorphs during reaggregation of adult sponge cells, when transdifferentiation occurs. Dissociation can be performed not only on the larva (see above §2.1) but also on the adult sponge (primmorphs). The development of primmorphs is accompanied by dedifferentiation, apoptosis, proliferation and differentiation again (figure 5*a*; [15]). At the stage of the formation of a new aquiferous system, we observed *ilborin* expression in the inner cells of a developing primmorph (figure 5*d*). Histological and ultrastructural observation of the primmorphs at the same stage revealed the development of new choanocyte chambers inside primmoprhs (figure 5*c*) and choanocyte differentiation accompanied by flagella formation (figure 5*b*). Thus, *ilborin* expression in the inner cells of the primmorph suggests that it is linked to the *de novo* formation of choanocyte chambers.

Ilborin presence was revealed by antibody staining in the form of prominent circles around choanocyte flagella [19]. Electron microscopy was used to check whether larval flagellated cells were morphologically similar to choanocytes. Special attention was paid to the apical parts of both cell types, which may posess specialized structures possibly containing ilborin.

Anteriolateral larval flagellated cells are elongated, narrow, columnar, 38–45 µm long and 2.6–3.0 µm wide in the nucleus region (figure 4*c*). They are characterized by a clear apical–basal polarity in the localization of cellular organelles and inclusions. The apical cell region contains an elongated nucleus (2.6 × 6.6 µm) with a small nucleolus, the flagellum and the flagellar apparatus (kinetid). Golgi complexes are present in the perinuclear cytoplasm in its apical part. The cytoplasm of the apical part of the cells includes abundant electron-transparent vesicles with an average diameter of 0.2 µm, oval mitochondria, small yolk granules and some spherical electron-dense granules. The basal two-thirds of these cells are filled with abundant large vitelline granules.

The cytoplasm of the choanocytes exhibits a marked apical–basal gradient in the organelle distribution (figure 4*d*). The nucleus is pyriform (4.5 × 2.6 µm), located distally, with an apical beak-like protrusion below the flagellum. A large Golgi apparatus encircles the nuclear apex. The apical cytoplasm includes numerous small vesicles with either electron-clear or electron-dense content. A few large vesicles and phagosomes are also present in this cell region. The mid part of the choanocyte cytoplasm contains most of the phagosomes and lysosome-like vesicles. The basal part of the choanocyte is devoid of organelles [22,23]. Though ilborin is apparently localized in the apical parts of the choanocytes (figure 4*b*), it cannot be attributed to any visible structures. Its cytoplasmic location agrees with *in silico* prediction. In the larva, *Hdu_ilborin* expression coincides with the protein localization at the anterior part of flagellated cells. *Ilborin* expression marks the choanocytes appearing during ordinary larva metamorphosis as well as during the development of primmorphs.

To understand the possible expression localization of ilborin orthologues, we examined single-cell RNA-seq data published for the sponge *Amphimedon queenslandica*, cnidarian *Nematostella vectensis* and ctenophore *Mnemiopsis leidyi* [24,25]. The *A. queenslandica* genome contains two paralogues of *ilborin*, Aqu2.1.23642_001 and Aqu2.1.10907_001; only the first was detected in metacell analysis of adult and larval cells of *A. queenslandica*. We found expression of Aqu2.1.23642_001 in two clusters of adult *A. queenslandica* cells, C43 and C24. Aqu2.1.23642_001 transcripts consist of about 20% of total unique molecular identifier in cells of the first cluster, C43; the authors, Sebé-Pedros *et al.*, attribute these cells to pinacocytes [24]. Cells in the second cluster, C24, are not so enriched with transcripts and their nature is unknown. Larval cells, overexpressing Aqu2.1.23642_001, belong to clusters C13 and C14. These cells are not described as any known larval cell type, but their molecular profile is similar to that of aspzincin cells of adult *A. queenslandica* [24]. The *N. vectensis* genome contains one *ilborin* gene (v1g246312 from genome v. 1, JGI), and its transcripts are detected in larval and adult polyp cells. Adult polyp cells expressing this gene belong to clusters C67 and C72 of the epidermal cells. Cells of larvae with v1g246312 overexpression fall into clusters of epidermis (C4) and progenitor/undifferentiated cells (C10–C14). Expression of *M. leidyi* orthologues of ilborin was not shown in these single-cell RNA-seq data. Based on these data, without spatio-temporal localization of gene expression we cannot postulate the functions of ilborin orthologues, but we can see that they are expressed in different basal invertebrates and their larvae.

### 2.4. Phylogeny of ilborin homologues

**Figure 6.**
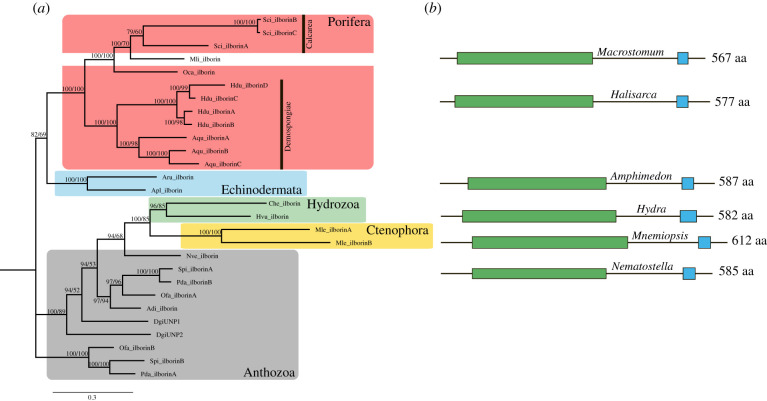
(*a*) Phylogenetic tree of ilborin orthologues from different phyla. Support values of posterior probabilities (top value) and bootstraps (bottom value) are displayed. Adi, *Acropora digitifera*; Apl, *Acanthaster planci*; Aqu, *Amphimedon queenslandica*;; Aru, *Asterias rubens*; Che, *Clytia hemisphaerica*; Dgi, *Dendronephthya gigantea*; Hdu, *Halisarca dujardini*; Hvu, *Hydra vulgaris*; Mle, *Mnemiopsis leidyi*; Mli, *Macrostomum lignano*; Nve, *Nematostella vectensis*; Oca, *Oscarella carmella*; Ofa, *Orbicella faveolata*; Pda, *Pocillopora damicornis*; Spi, *Stylophora pistillata*; Sci, *Sycon ciliatum*. (*b*) Domain structure of representative ilborin orthologues for each class. Green, aldolase-type TIM barrel; blue, EF-hand domain; polypeptide length is given as the number of amino acid residues.

The search against transcriptomic and genomic databases of different organisms, from choanoflagellates to vertebrates, with ilborin sequence as a query produced a number of unidentified homologues. They were found in Porifera, Cnidaria, Ctenophora, Platyhelminthes and Echinodermata (figure 6*a*; electronic supplementary material, files S10 and S11). No homologues were observed in Choanoflagellata or chordates. Two bacterial proteins with the same domain structure and a significant *e*-value were found in the cyanobacteria *Moorea producens* and *Okeania hirsuta*. In Bayesian and maximum-likelihood (ML) analyses, the bacterial sequences fall into a sister clade to the metazoan ones and were used to root the tree (figure 6*a*). Ilborin homologues are present in three classes of Porifera: Demospongiae, Calcarea and Homoscleromorpha. The well-studied sponges *A. queenslandica* (Demospongiae) and *Sycon ciliatum* (Calcarea) have three paralogues each. Among cnidarians, the orthologues were present in Hydrozoa (*Hydra vulgaris*, *Clytia hemisphaerica*), Scyphozoa (*Aurelia aurita*) and numerous Anthozoa (*N. vectensis*, *Orbicella faveolata*, *Pocillopora damicornis* and others). Interestingly, both ctenophore sequences were placed inside a shared cnidarian branch. Our search in numerous datasets of planarians, nematodes and trematodes did not reveal any homologues. The only protein identified in Lophotrochozoa was found in *Macrostomum lignano*, a new model species of Platyhelminthes [26]. Surprisingly, it was placed inside the Porifera clade with high support. The lengths of all orthologous proteins (560–610 aa residues), their conserved domain composition, and their relative positions were congruent with ilborin structure (figure 6*b*). So, ilborin homologues are conserved in several different invertebrate groups.

### 2.5. EF-hand domain analysis

**Figure 7.**
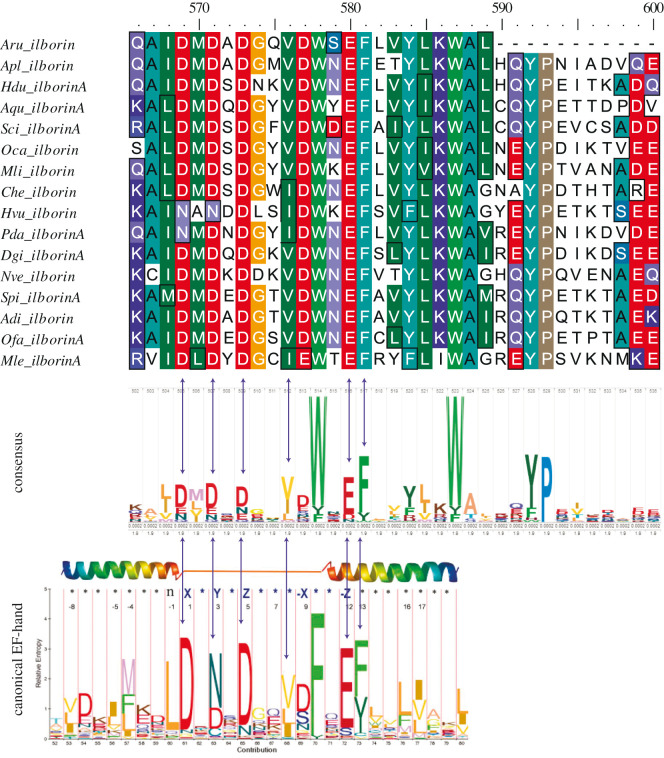
Position of conserved amino acids responsible for Ca-binding in the EF-hand domain. Top: alignment of EF-hand of different ilborin orthologues. Middle: consensus generated with HMM Logo from top alignment. Bottom: position of conserved residues in the loop and second helix of canonical EF-hand (marked with arrows). Residues X, Y, Z and -Z (numbered 1, 3, 5 and 12, respectively) are responsible for Ca^2+^ binding (modified from [46, Fig. 2]).

EF-hand domain is a characteristic feature of all ilborin orthologues. We generated HMM LOGO of the consensus EF-hand domain (figure 7) and compared it with the Ca-binding sites of known EF-hand proteins. The relative position of aspartate, glutamate and phenylalanine residues in the consensus corresponded to that of amino acid residues in EF-hand responsible for calcium binding (residues 1, 3, 5 and 12 in terms of standard numeration of residues in EF-hand) (figure 7) [27,28].

**Figure 8.**
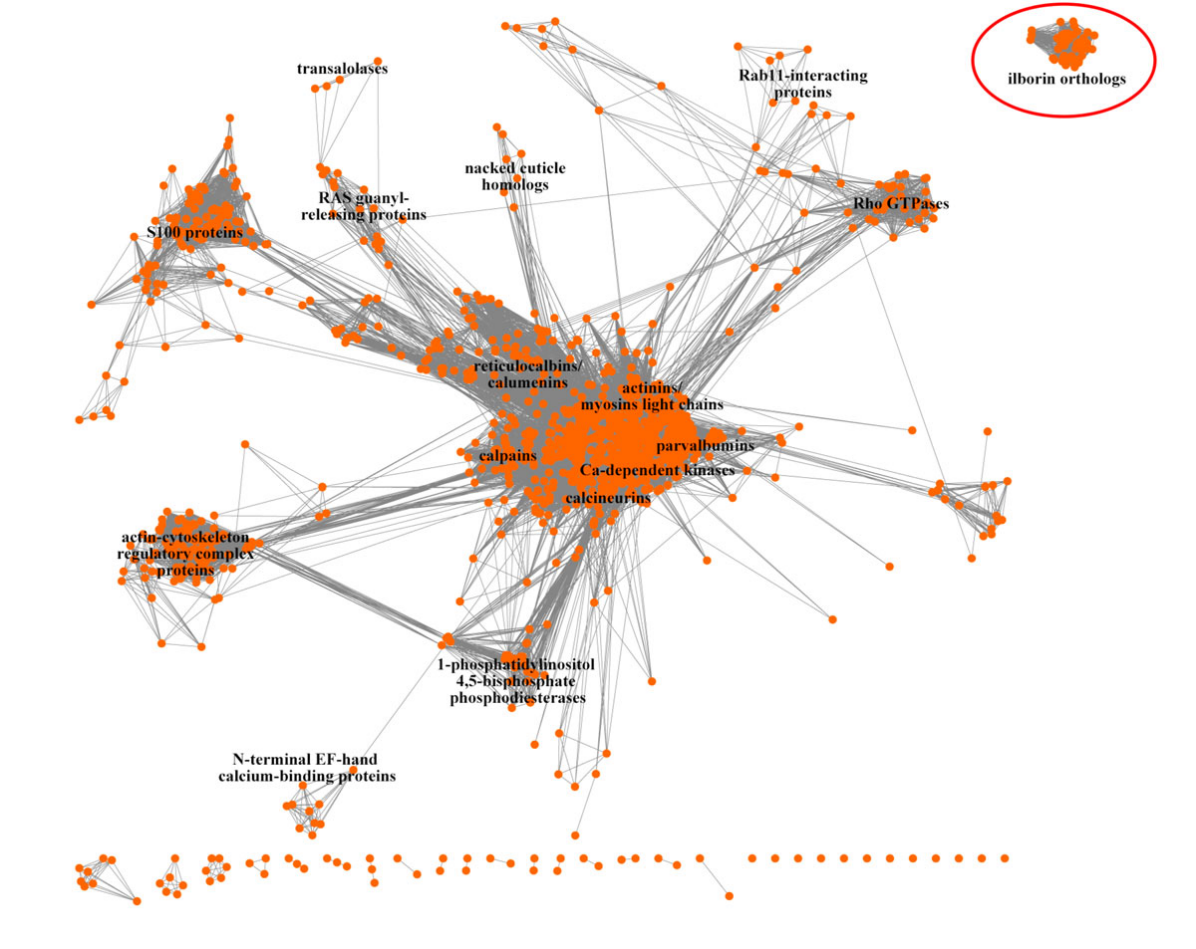
Sequence similarity network of EF-hand-containing proteins generated by all-versus-all BLAST and visualized with Cytoscape. Each orange circle is an entry (EF-hand protein sequence); grey lines are edges demonstrating percentage of identity in alignment. Nodes clustered with the Markov cluster (MCL) algorithm. Some major EF-hand protein subfamilies labelled. Ilborin orthologues cluster is at the top right; singletons are below.

A large part of the molecule is occupied by the TIM barrel domain. We suggested that this part of the ilborin sequence might be similar to other EF-hand-containing proteins. In order to check this hypothesis, we compared full-length EF-hand-containing proteins from the Swiss-Prot database and ilborin orthologues by all-versus-all basic local alignment search tool (BLAST). Based on the BLAST results, a sequence similarity network (SSN) was built and clusterized (figure 8) [29]. Each edge (line connecting two nodes) means that these nodes (entry sequences) had reciprocal BLAST similarity with an *e*-value above the threshold. The similarity between the two sequences became visible. Thus, the cluster, which is a group of nodes located close to each other, represents the same subfamily. About 95% of homologues of Ca-binding proteins such as calmodulin, tenascin, parvalbumin, Ca-dependent protein kinases, calpain, hippocalcin, alpha-actinin and others cluster together into corresponding subfamilies (figure 8). About 95% of calcium-binding proteins from Swiss-Prot (1620 of 1684) were included in a common network; their similarity level is above the threshold of 50% identity in amino acid residues. Another fraction of 64 proteins formed a number of little clusters of two to eight sequences. Ilborin homologues form a separate cluster with no links to any other subfamily of EF-hand proteins. All ilborin orthologues are predicted to be able to bind Ca^2+^ ions, and they definitely form a separate subfamily among EF-hand proteins with proved Ca-binding. This is a new subfamily, which has never been described before to our knowledge.

### 2.6. Remote similarity of ilborins

A prominent difference between ilborin orthologues and other EF-hand-containing proteins prompted our search for their remote similarity to other proteins with the HHblits algorithm. An alignment of four Hdu_ilborin paralogues was used as query in HHblits search. It produced a full-length significant hit (*e*-value: 8.8e−74; identity: 39%) with pyruvate carboxyltransferase domain-containing protein of *Hondaea fermentalgiana* (Labyrinthulomycetes) (A0A2R5GS87) (electronic supplementary material, file S12). At the same time, no conserved domains in Hdu_ilborins A–D were identified by Pfam search, where pyruvate carboxylase domain was an insignificant hit. We analysed further a part of the sequence corresponding to the supposed enzymatic domain (1–355 aa residues based on Hdu_ilborin A). HHpred search based on the alignment of all proposed enzymatic domains of ilborins produced significant hits with homocitrate synthase (2ZTJ_A, 2.9*e*-21) and hydroxymethylglutaryl-CoA lyase (1YDN_D, 2.7*e*-17). Two metal ion-binding sites (His205, His207) shared by all ilborins are also conserved in all these enzymes. A substrate-binding site (Arg35) and a third metal-binding site (Glu36) are present in all ilborin orthologues except those with shortened sequences (*Aru*_ilborin, *Acu*_ilborinA, *Che*_ilborin) (electronic supplementary material, file S8). The Glu36 position is identical to that of homocitrate synthase and is substituted by Asp in hydroxymethylglutaryl-CoA lyase. The enzymes identified are widespread in both prokaryotes and eukaryotes [30–32]. Their functions are associated with pyruvate and acetyl-CoA metabolism [33,34] and ketogenesis [35]. Both these processes are tightly bound to the energy supply of the cell [36,37].

So, ilborin is a new member of the Ca^2+^-binding proteins family. It is the major protein of flagellated cells of sponge larvae, and its expression marks the future and the developing choanocytes. Ilborin domain structure and remote similarity suggest its involvement in the energy turnover in the cell.

## 3. Discussion

### 3.1. Ilborin structural features

At the first step of the S-antigen identification, BLAST search through all databases did not show any similarity with any described proteins. We found a number of previously undescribed orthologues in different invertebrate phyla but not in ancestral choanoflagellates. These orthologues have not been annotated, the information about them being limited to the name of the organism from which the transcriptome or the genome of the sequence was isolated. *Halisarca dujardini* ilborins and all the sequences collected from public databases possess an identical domain architecture: they contain an EF-hand domain and an aldolase TIM barrel domain (figure 3). They also show a high level of sequence similarity. Many of the species studied have more than one paralogue in their genome: in 13 species, 25 different ilborins were found. The large number of similar sequences suggests that they belong to a newly defined family of metazoan proteins.

Substantial differences in 5′ and 3′ UTRs seem to indicate that four *ilborin* paralogues are encoded by different genes. Other species also have more than one *ilborin* transcript or even gene (for species for which a sequenced genome is available). These features point to different genes rather than allele variants or products of differential splicing. While *Hdu_ilborin A–D* cDNAs have been cloned and sequenced, the sequences from other invertebrates are hypothetical mRNA/protein-coding sequences (CDSs) that are yet to be characterized. Still, we suppose that these RNAs arise from different genes.

Two putative conserved domains were identified in the amino acid sequence based on comparison with the databases: the aldolase TIM barrel domain and the EF-hand domain. Aldolase TIM barrel is a protein superfamily with a widespread and universal TIM barrel protein fold. It consists of eight α-helices and eight parallel β-strands. This type of structure is considered to be extremely ancient, being present in all three domains of life. The authors of a study based on functional and taxonomic diversity concluded that Tim barrel superfamilies emerged early in evolution and subsequently underwent wide functional expansion [38]. The TIM barrel fold is currently known to be present in the structure of approximately 10% of all described enzymes [39,40]. Thus, the presence of the TIM barrel domain in ilborins highlights their similarity to enzymatic proteins.

Another prominent feature of ilborins is the EF-hand domain. We used reciprocal BLAST and constructing an SSN to establish the relationship between ilborin and other EF-hand proteins (figure 8). Full-length sequences were used in the analysis, so that the similarity throughout the protein and the presence of other conserved domains could be taken into account. The analysis showed that ilborin orthologues formed a separate group without a high similarity with any class of EF-hand proteins.

### 3.2. Putative ilborins’ functions

Ilborin homologues have high sequence similarity to each other and their domain architecture is conserved across several groups of Metazoa. This observation suggests that ilborin proteins have a common function. The predicted EF-hand domain is at the C-terminal end of all ilborins and their homologues. The EF-hand unit comprises a conserved motif of a helix–loop–helix structure that binds a single Ca^2+^ ion. The loop consists of 12 residues with the pattern X∗Y∗Z∗∗∗-X∗∗-Z. The residues X, Y, Z, -X and -Z participate in binding Ca^2+^, while intervening and less conserved residues are marked by asterisks. Asp or Asn is usually found at the X and Y positions; Asp, Asn or Ser at the Z and -X positions; and Glu at the -Z position. The EF-hand domain is only 29 residues long, and the loop consists of residues 10–21. Residues 15 and 17 inside the loop, Gly and Ile, respectively, are highly conserved. There are EF-hand domains that do not bind calcium [41]. We performed a deep analysis of ilborins' EF-hand domains in order to check their functional ability.

In quiescent cells, proteins with EF-hands are in an apoprotein form. When the cytoplasmic level of Ca^2+^ ions increases, they bind Ca^2+^ and change their conformation. Сonformation change is a feature of proteins involved in signal transduction of calcium signalling as a secondary messenger [42]. EF-hands can be present in proteins alone or together with other known domains. In most cases EF-hands are found in pairs, the EF-lobe, which is a unit of evolution. Although all EF-hand proteins are inferred to have evolved from a single precursor helix–loop–helix domain by gene duplication and fusion, a single EF-hand is so short that it cannot be ruled out that it arose de novo several times [41,43].

All ilborin orthologues have a single putative calcium-binding domain consisting of one EF-hand. The residues that participate in Ca^2+^ coordination are conventionally used as a reference frame to analyse calcium-binding ability. EF-hand loops of ilborin homologues are composed of highly conserved key amino acid residues of the canonical domain structure. We showed that these domains shared a high level of conservation in the residues that participate in the chelation of calcium ions. The EF-hand of ilborin contains the main residue pattern characteristic of the calmodulin canonical EF motif, the DxDxDG [44,45]. This pattern is absent in pseudo-EF-hand loops from the most recent vertebrate S100 family of proteins [46]. It is highly probable that the ilborin EF-hand possesses Ca^2+^-binding capacity. Our bioinformatics analysis strongly suggests that the ilborin family may be a new type of Ca^2+^-binding proteins (figure 8). Nevertheless, experimental confirmation of the actual Ca^2+^-binding is required along with the evidence at the sequence conservation level.

Ilborin and its homologues possess a remote similarity to different enzymes owing to the presence of the aldolase TIM barrel domain. In general, the aldolase TIM barrel superfamily is most often associated with sugar metabolism and energy generation [38]. TIM barrel-based enzymes are very diverse, and ilborins are most close to several specific representatives of this superfamily. Functionaly, the pyruvate carboxyltransferase domain is a part of the enzyme pyruvate carboxylase, which produces oxaloacetate for the Krebs cycle. This means that pyruvate carboxyltransferase is involved in oxygen- dependent ATP production. It is also involved in gluconeogenesis [47,48]. Hydroxymethylglutaryl CoA lyase is a key enzyme in ketogenesis, serving as an alternative energy source in energy-demanding tissues [37]. Ketone bodies produced in ketogenesis can pass through the plasma membrane of different cells and supply energy through NADH production [49]. At the same time, homocitrate synthase is involved in lysine biosynthesis [34]. Thus, two of the three enzymes that show remote similarity to ilborins are related to energy turnover.

Sponge ilborins were originally revealed in larval flagellated cells (figures 2 and 4). During metamorphosis these cells rapidly, within hours, disrupt cell junctions, dedifferentiate, move and change their position from external to internal. This cell type is supposed to be energy-demanding because of their active movement. So, the similarity of ilborin to energy-related enzymes makes biological sense. While homocitrate synthase and pyruvate carboxyltransferase are usually mitochondrial enzymes, hydroxymethylglutaryl CoA lyase has cytosol [35] and peroxisomal isoforms [50]. Ilborins are predicted by *in silico* methods to be cytosolic proteins, which is in agreement with experimental observations (figure 4). Transmission electron microscopy of flagellated cells shows that there are few mitochondria near the apical surface of flagellated cells, where the basal body resides (figure 4*d*) [22,51]. Ilborin is localized in the cytoplasm of flagellated larval cells and remains there when the cells lose flagella during metamorphosis, as was shown by immunostaining [19]. In newly formed choanocytes of the juvenile sponge ilborin is concentrated around the arising flagella. The absence of mitochondria or any visible structures around the flagellar base in juvenile sponge choanocytes (figure 4*d*) is in agreement with bioinformatic prediction. We believe that the unique protein composition of larval flagellated cells (figure 2) suggests their preparation for rapid and substantial changes evoked by transdifferentiation. If this is the case, the presence of ilborin in flagellated cells can be explained by its anticipatory expression and storage for subsequent use in apical cytoplasm structure formation. Probably, the cytoplasmic level of Ca^2+^ ions increases during metamorphosis, and ilborin binds Ca^2+^ and changes its conformation, acquiring enzymatic capacity. Calcium-dependent regulation of metamorphosis was demonstrated in different marine phyla, including sponges and polychaetes [52,53]. A novel calcium-binding protein has recently been described in Ambulacraria [54], and the regulatory role of calcium ions may be even broader than known today. There are no data on protein/transcript localization of ilborin homologues in other invertebrates.

## 4. Conclusion

In this work we report a new protein, ilborin, and its orthologues. Functional studies of ilborin lie ahead. Full-length ilborin RNA has already been cloned and could be expressed, which would open the way to the testing of protein properties. The search for remote similarity suggests that ilborin is involved in energy turnover. It is known that EF-hand can change entire protein conformation upon Ca-binding [55,56]. Possible ilborin involvement in the energy cycle could be regulated in the same way, through changes in cytoplasmic Ca^2+^ concentration, which occur during metamorphosis and/or preparation to it. Ilborin is definitely involved in metamorphosis and sponge cell transdifferentiation. A broad distribution of ilborin orthologues among invertebrates and a large amount of ilborin in flagellated larval cells suggest that this protein plays an important role in the living cells of invertebrates.

## 5. Methods

### 5.1. Specimen collection

Reproducing specimens of *H. dujardini* were collected in the Chupa Inlet near Sredniy Island, 33°05′ E, 66°15′ N (Kandalaksha Bay, White Sea) from a depth of 1.5–5 m from June to August. The sponges attached to the alga *Fucus vesiculosus* were transported to the laboratory of the Educational and Research Station ‘Belomorskaia’ of St Petersburg State University (SPbU) and maintained in aquariums at 14°C. Spontaneously released larvae were collected manually twice a day by means of a Pasteur pipette.

### 5.2. Cell separation and protein identification

The larvae of *H. dujardini* were collected with a Pasteur pipette and 40 µm Falcon cell strainer (cat. no. 352340) and transferred to artificial Ca-free seawater (ASW) containing 0.3 M sodium chloride, 20 mM potassium chloride, 45 mM EDTA and 10 mM Tris-HCl, pH 7.7 [19]. This salt composition, corresponding to an osmolarity of 740 mOsM, is equal to 27‰, which is the salinity of seawater at the site where the sponges were collected. Dissociation of cells, observed under a stereomicroscope, took place in 3–5 min.

A discontinuous density gradient was manually prepared with Percoll (GE Healthcare; [57]) steps of 10, 25, 35, 40 and 45%. An appropriate volume of Percoll was mixed with artificial Ca-free seawater to make the solution, after which aliquots of 1.5 ml were overlaid sequentially in a centrifuge tube. A suspension of dissociated cells (1.5 ml) was overlaid on top of the 10% layer of Percoll solution. The tubes were centrifuged in a swing rotor at 800*g* for 10 min with extremely gentle starting and stopping. Each cell layer was collected by gentle aspiration. Each fraction was diluted with Ca-free seawater to reduce the Percoll concentration, and cells were collected by centrifugation. The pellet was resuspended and boiled in loading buffer and subjected to 10% SDS-PAGE [58]. The gels were stained with Coomassie Brilliant Blue, and a major band with the expected molecular weight (see Results) was excised. MS analysis was performed in the Proteomics Unit of theInstitute of Biotechnology, University of Helsinki, Finland. Briefly, the protein was digested in-gel with trypsin, the peptide mixture was purified on a C18-column and applied to an LC-(H)ESI Orbitrap Elite Hybrid mass spectrometer (Thermo Scientific). Tandem MS/MS spectra were deconvoluted with Xcalibur software (Thermo Scientific) and searched against the database of the *in silico-* translated transcriptome of *H. dujardini* [20]. Reciprocal tBLASTn comparison of the initially identified ilborin against the *H. dujardini* transcriptome revealed the presence of three additional sequences with a high similarity level.

### 5.3. Primmorphs

Only non-reproducting sponge specimens, collected from late August to October, were used for reaggregation experiments. Sponge tissues were dissociated by mechanical squeezing through 50 µm nylon mesh into vessels containing sterile seawater (FSW). FSW was used in the dissociation procedure and during subsequent cell cultivation to avoid additional contamination. Water was sterilized with 0.22 µm Millex-GP syringe filter units (Merck Millipore). The cell concentration in the suspensions was determined by using a haemocytometer. Subsequently, the cell suspensions were diluted with FSW to concentrations of 1–3 × 10^7^ cells ml^−1^. The cell cultures were maintained in 30 mm plastic Petri dishes (5 ml of cell suspension per dish) in FSW at 8–10°С. An orbital shaker (70 r.p.m.) was used to intensify the aggregation at the initial stage of the reaggregation process: the cultures were maintained on the shaker during the first hour after tissue dissociation. Half of the FSW was replaced with a fresh portion every 48 h. Each cell culture was checked and photographed daily during the whole period of cultivation using a Leica M165FC stereomicroscope equipped with a digital camera (Leica DFC420C) and the application Leica LAS Store and Recall v. 4.2. For whole-mount *in situ* hybridization (WMISH), developing primmorphs were fixed overnight at 4°C in MEMFA (4% paraformaldehyde, 2mM ethylene glycol-bis(2-aminoethylether)-*N*,*N*,*N*′,*N*′-tetraacetic acid (EGTA) in 0.1 M 4-morpholinepropanesulfonic acid (MOPS)) and then gradually dehydrated in an ethanol series and stored in 70% ethanol at −20°C.

### 5.4. Cloning and *in situ* hybridization

cDNA was synthesized with MMLV reverse transcriptase (Evrogen) from total RNA extracted from larvae with TRIzol reagent (Thermo Scientific). Primers specific for the 543 bp fragment were designed based on full-length transcript from the *H. dujardini* transcriptome (F: 5′-CAGTTCAACGACGAGATTTG-3′; R: 5′-CCTGAAAGATGTGCCTTTG-3′). The fragment was cloned in pAL-2T vector (Evrogen). Inserts were verified by Sanger sequencing and used for probe synthesis. Antisense digoxigenin-labelled RNA probe was made by *in vitro* transcription with DIG RNA labelling mix (Roche) and an appropriate RNA polymerase (Thermo Scientific). WMISH of the larvae was performed as described [20]. Alkaline phosphatase-labelled probe was visualized with the NBT/BCIP colorimetric substrate system (Roche).

To confirm the existence of different transcripts, cloning was performed as follows. cDNA was synthesized with random primers from total RNA. Primers were designed in such a way as to amplify CDS and at least part of both 5′ and 3′ UTRs (electronic supplementary material, file S2). PCR was carried out with theTersus Plus PCR kit (Evrogen). PCR products were cloned in pAL2-T plasmid (Evrogen) and subjected to Sanger sequencing.

### 5.5. Microscopy

For transmission electron microscopy (TEM) specimens were fixed with 2.5% glutaraldehyde and post-fixed with 1% OsO_4_. Specimens were embedded in Araldite (Sigma-Aldrich). Ultrathin sections (60–80 nm) were cut with an Ultramicrotome PowerTome XL, equipped with a Drukkert 45° diamond knife, and contrasted with uranyl acetate. Ultrathin sections were studied under a Zeiss-1000 (Carl Zeiss) transmission electron microscope. The images were processed using ImageJ software (FiJi).

### 5.6. *In silico* sequence analysis and phylogeny

The average molecular mass and isoelectric point were calculated with ProtParam [59] on the ExPASy server (https://www.expasy.org/). Subcellular location was predicted with SignalP 5.0, SCL-Epred [60] and WoLF PSORT [61]. Protein backbone dynamics were predicted with DynaMine [62]. Attribution to protein superfamilies and the Ca-binding site were predicted with InterPro 79.0 [21].

For remote similarity search we used both HHblits [63] and HHpred [64] algorithms. In HHblits alignment of the four paralogues, the sequence of ilborin from *H. dujardini* (*Hdu_*ilborin) was queried in the UniRef30_2020_03 database. For HHpred search, all related proteins were aligned with MAFFT (v. 7.402) and corrected manually. Part of the alignment 1–355 aa based on Hdu_ilborin A corresponding to the proposed enzymatic domain was queried in the PDB database (PDB_mmCIF70_29_May). Positions of active site residues were assessed based on experimentally validated interaction with the substrate or a metal ion [31,65].

Unannotated homologues were identified in genomic and transcriptomics datasets with tBLASTn at the NCBI server, Fungal genome resource of the Joint Genome Institute (https://mycocosm.jgi.doe.gov/mycocosm/home), Ensembl Metazoa server (https://metazoa.ensembl.org), Compagen server (http://www.compagen.org/), *M. lignano* genome project (http://www.macgenome.org/), *Caenorhabditis elegans* server (https://wormbase.org/), *Platynereis* server (http://pdumbase.gdcb.iastate.edu/platynereis/) and Planarians database server (http://planmine.mpi-cbg.de/). Conserved domains in identified sequences were determined with InterProScan (v. 5.44–79.0). Proteins of other invertebrates were aligned with those from *H. dujardini* using MAFFT (v. 7.402) and corrected manually. Maximum-likelihood phylogenetic trees were estimated using the IQ-TREE web server [66]. Statistical support was estimated by performing 100-bootstrap replicates. Bayesian analyses were performed with MrBayes v. 3.2.7, using the Le & Gascuel (LG) + *Γ*+I model of evolution, with four chains, a subsampling frequency of 100, and four parallel runs, each 500 000 generations. The best-fit model for our set of proteins was chosen using ModelTest-NG (v. 0.1.5). Bayesian posterior probabilities were used to assess the confidence values of each bipartition. The analysis was carried out using computational resources provided by the Resource Centre ‘Computer Centre SPbU’ (www.cc.spbu.ru). Trees were visualized with FigTree 1.4.4 software.

Ilborin sequences were compared with other EF-hand proteins as follows. Annotated EF-hand proteins were obtained from the Swiss-Prot database (Prosite: PS50222). Ilborin orthologue sequences were joined to the Swiss-Prot entry, and all-by-all BLAST was performed in order to obtain the similarities between the sequence pairs and to calculate edge values for the generation of the SSN. Network files were created with EFI-EST (Enzyme Similarity Tool, [29]) under default settings, as the node for each entry and the edge for similarity or identity between entries. The network was visualized and analysed using Cytoscape 3.8.0 [67]. Markov clustering (MCL) was performed with clusterMaker (v. 1.11), a Cytoscape plug-in in the Markov Clustering Algorithm [68]. HMM LOGO of the EF-hand domain was generated with the hmmsearch web-tool based on one ilborin paralogue from each analysed species owing to low sequence divergence.

## Data accessibility

All data generated or analysed during this study are included in this published article and its supplementary information files. DNA sequences: Genbank accession nos MT559266–MT559269.
